# Biomass fuel use and the exposure of children to particulate air pollution in southern Nepal

**DOI:** 10.1016/j.envint.2014.01.011

**Published:** 2014-05

**Authors:** D. Devakumar, S. Semple, D. Osrin, S.K. Yadav, O.P. Kurmi, N.M. Saville, B. Shrestha, D.S. Manandhar, A. Costello, J.G. Ayres

**Affiliations:** a UCL Institute for Global Health, 30 Guilford St., London WC1N 1EH, UK; b University of Aberdeen Scottish Centre for Indoor Air, Division of Applied Health Sciences, Royal Aberdeen Children's Hospital, Westburn Road, Aberdeen AB25 2ZD, UK; c Mother and Infant Research Activities, Thapathali, PO Box 921, Kathmandu, Nepal; d Clinical Trial Services Unit and Epidemiological Studies Unit, University of Oxford, Richard Doll Building, Old Road Campus, Roosevelt Drive, Oxford OX3 7LF, UK; e Institute of Occupational and Environmental Medicine, University of Birmingham, Edgbaston, Birmingham B15 2TT, UK

**Keywords:** GPS, Global Positioning System, LOD, limit of detection, PM, particle mass, TWA, time-weighted average, Child health, Particulate matter, Exposure modeling

## Abstract

The exposure of children to air pollution in low resource settings is believed to be high because of the common use of biomass fuels for cooking. We used microenvironment sampling to estimate the respirable fraction of air pollution (particles with median diameter less than 4 μm) to which 7–9 year old children in southern Nepal were exposed. Sampling was conducted for a total 2649 h in 55 households, 8 schools and 8 outdoor locations of rural Dhanusha. We conducted gravimetric and photometric sampling in a subsample of the children in our study in the locations in which they usually resided (bedroom/living room, kitchen, veranda, in school and outdoors), repeated three times over one year. Using time activity information, a 24-hour time weighted average was modeled for all the children in the study. Approximately two-thirds of homes used biomass fuels, with the remainder mostly using gas. The exposure of children to air pollution was very high. The 24-hour time weighted average over the whole year was 168 μg/m^3^. The non-kitchen related samples tended to show approximately double the concentration in winter than spring/autumn, and four times that of the monsoon season. There was no difference between the exposure of boys and girls. Air pollution in rural households was much higher than the World Health Organization and the National Ambient Air Quality Standards for Nepal recommendations for particulate exposure.

## Highlights

• Air pollution exposure (respirable PM) was estimated for children in Nepal.
• Gravimetric and photometric microenvironment sampling was conducted.
• Total sampling was repeated three times over a year and totaled over 2500 hours.
• The 24-hour time weighted average was 168 µg/m3.
• There was substantial variability from season to season and by fuel type.
• There was no difference in exposure between boys and girls.

## 1 Introduction

Indoor air pollution is a major cause of ill-health in low-income countries. It is mostly due to the burning of biomass fuels (also referred to as “solid fuels”), a group of organic materials – particularly wood, dung, straw, and charcoal – used as a source of heat and light (Rehfuess, 2006). It is estimated that between one-third and half of the world's population use biomass as a source of energy because it is readily available and usually cheap (Torres-Duque et al., 2008). Globally, solid fuel use is estimated to cause 3.5 million premature deaths per year, around one million of which are attributed to acute respiratory infections in young children (Lim et al., 2012; Murray et al., 2012). The deaths occur predominantly in poorly resourced settings where an increased susceptibility to illness coexists with high levels of pathogens in the environment and reduced access to healthcare. As well as increased mortality, household cooking with solid fuels accounts for 4.3% (95% CI: 3.4 to 5.3) of Disability Adjusted Life Years lost worldwide (6% for children under 5 years old), while ambient air pollution accounts for a further 3.1% (95% CI: 2.7 to 3.4). These figures make indoor air pollution the third leading contributor to global disease burden, and the highest in South Asia (Lim et al., 2012). There is strong evidence linking solid fuel use to chronic obstructive pulmonary disease (Kurmi et al., 2010), pneumonia in children under 5 (Dherani et al., 2008), lung cancer (Kurmi et al., 2012), and tuberculosis (Sumpter and Chandramohan, 2013). There is also weaker evidence for a link with low birthweight (Pope et al., 2010) (Shah and Balkhair, 2011), anemia and stunting (Fullerton et al., 2008; Kyu et al., 2009; Rehfuess, 2006).

Incomplete combustion of biomass fuels in poorly ventilated houses produces domestic levels of airborne particles hundreds of times higher than commonly encountered outdoors (Fullerton et al., 2008). Indoor air concentrations of PM_10_ (particles with median diameter less than 10 μm) can be up to 10 000 μg/m^3^ during cooking (Rehfuess, 2006).

Biomass fuel usage is very common in Nepal, with estimates of use in 75% of households (Statistics, 2012), particularly in poorer areas outside the major cities. The burning of biomass in Nepal has been shown to adversely affect lung function in young adults (Kurmi et al., 2013) and exacerbate respiratory disease in children (Bates et al., 2013). Despite its high prevalence and adverse health effects, most research takes fuel usage as a proxy for true exposure. In this study, we sampled the respirable fraction of particle mass (PM_4_) for children aged 7 to 9 years in the microenvironments in which they spent time. We also collected data on fuel usage, household characteristics and children's time–activity patterns, to produce a time-weighted average (TWA) exposure.

## 2 Materials and methods

The study was part of a larger follow-up of children born after a randomized controlled trial in which pregnant women were allocated to multiple micronutrient or iron and folic acid supplements (Osrin et al., 2005). We attempted to find all the children from the trial at seven to nine years of age. Particulate matter concentrations were measured in a subsample and the data were used to model the likely exposure for all children based on household fuel usage and time–activity information. A 24-hour TWA exposure estimate was created for each child in relation to respirable particulate (< 4 μm median aerodynamic diameter — PM_4_). Sampling was carried out from December 2011 to December 2012. Questionnaire data were collected for all children in the cohort whom we were able to find.

### 2.1 Setting

The study was predominantly carried out in Dhanusha district in the Terai region that makes up the southern half of Nepal, bordering India. Nepal ranks 157th out of 186 countries in the Human Development Index and average life expectancy is 69 years (UNDP, 2013). A plain district with a population of 760 000, Dhanusha's economy centers on agriculture. The urban samples were taken in the district capital, Janakpur, which houses about one-eighth of the district population (Central Bureau of Statisics, 2012a). There are few asphalted roads, even in the city. Mechanized traffic consists mostly of motorbikes and small numbers of cars, tractors, trucks and buses.

### 2.2 Sampling strategy

Samples were taken at the following locations:

• Bedroom. A Casella Apex gravimetric sampler was placed in the room where the child slept, set to sample from late afternoon and collected first thing the following morning. For the first season we chose to sample in 40 houses, corresponding to 5% of the total expected sample number.
• Veranda. A TSI DustTrak monitor was placed in the veranda, approximately equidistant from the inside of the building and the outside. Sampling was done in the evening to coincide with the time that the child was normally on the veranda.
• Kitchen. An Apex sampler was placed in the kitchen about 1 m away from the stove for the period of cooking, and also when there was no cooking for a three-hour period (at least 1 h after cooking had ceased). The DustTrak was also used to collect seven 12-hour samples to look at changes in air pollution concentration over time.
• School. Four urban and four rural schools were chosen according to their accessibility and willingness to participate. Consent was taken from school principals prior to sampling. An Apex sampler was placed in a classroom chosen by the principal, during school hours, which varied from school to school and by season. Care was taken not to place it close to the door, windows, or blackboard. All classrooms were on the ground floor.
• Outdoors. Outdoor samples were taken by members of staff, close to their homes in eight rural and urban areas. An Apex sampler was kept in the garden or compound, as far away as possible from the house or adjacent houses.

To take account of seasonal variation in air pollution levels, we repeated the measurements three times over a year. Samples were taken during the winter season (December to March), the monsoon season (June to September), and the hotter spring and autumn seasons (April, May, October and November). We stratified our sample by urban or rural location as we thought the dust and traffic and the close proximity of air pollution from neighbors would make these locations different. We also stratified bedroom samples by ceiling type as different roof types were believed to allow different degrees of ventilation. We stratified the kitchen samples by the type of fuel used: biomass and non-biomass. We attempted to make the sample representative of all the children in our study. We chose houses to sample in by randomly ordering the first 100 children seen in the larger follow-up study, and proceeding down the list until the required number in each stratum was found. After assessing the results from the first season, the sampling schedule was adapted. Due to the lack of variation by roof type, the number of bedroom samples was reduced and the number of outdoor and kitchen samples increased. Sampling times and duration are shown in Table 1.

### 2.3 Time activity

During a pilot phase, field team members visited 40 families with children aged seven to nine years to establish the main locations that children spent their time in. The questionnaire was then administered to all 851 children in the cohort. A parent or guardian was taken through an “average” school day and asked to say where the child would be for most of the time in half-hour blocks over a 24-hour period. Children divided their time between five locations: bedroom/living room, kitchen, veranda, in school and outdoors. Periods in the kitchen were subdivided into time when cooking was taking place and time when it was not. A table was created for each child, summarizing the amount of time in each pre-determined location.

### 2.4 Gravimetric sampling

Gravimetric sampling was conducted in accordance with the “Methods for Determination of Hazardous Substances (MDHS) no. 14/3 (Health and Safety Executive, 2000) guidelines” (Health and Safety Executive, 2000), using the Casella Apex gravimetric sampler (Casella, Bedford, UK). New glass fiber 37 mm filters (Casella, Bedford, UK) were weighed on a Sartorius balance (Sartorius Ltd., Epsom, UK) accurate to 0.00001 g, and calibrated annually. The filters were pre-weighed in the UK as close as possible to departure for Nepal. Post-sample weighing was done in four batches over a nine month period, determined by the logistics of travel to the field site. Filters were kept in the laboratory overnight to acclimatize, and then weighed twice over two consecutive days and the average calculated. The filter was discarded if the two weights differed by more than 100 μg. Each filter was placed in a plastic “filter keeper” (SKC Ltd., Dorset, UK).

Air sampling was conducted using an Apex air pump attached to a cyclone sampling head (Casella, Bedford, UK) to collect respirable sized particulate in accordance with MDHS guidelines (Health and Safety Executive, 2000). Flow rates were set to 2.2 L/min using a portable flow meter (Casella Ltd. Rotameter, range 0.5 to 5 L/min). This was calibrated in turn with a Bios Dry Cal DC-Lite Primary flowmeter prior to taking it to Nepal. The sampling head was placed at about 1.1 m from the ground and attached to a portable Leicester stadiometer. This height was chosen because it was thought to be approximately the height of the child's mouth and nose. Time was recorded from data collectors' mobile phones, which was synchronized weekly. The sample was discarded if the total sampling time differed by more than 5% from the time recorded on the Apex pump. After sampling, each filter was placed in a sealable plastic filter keeper and then in an airtight box, and taken to the United Kingdom where it was reweighed using the same balance and weighing protocol. Filters were examined and discarded if loss of material was thought to have occurred. One in ten filters were used as field blanks to correct for changes in filter weight.

### 2.5 Photometric sampling

A TSI DustTrak II 8530 monitor (TSI Inc., St. Paul MN, USA) was used to measure particulate concentrations in the veranda and some of the kitchen microenvironments. This device provided 1-minute resolution of respirable dust concentrations. Sampling was performed at 1.7 L/min using a Dorr-Oliver cyclone attachment (TSI Inc., St. Paul MN, USA). The DustTrak was zero calibrated, pre-programmed to run for the appropriate time, and placed in the location. Data were downloaded via TSI Trakpro software.

### 2.6 Analysis

Concentration was calculated using formula (1). After reweighing the filters, the average pre-weight was subtracted from the average post-weight. This was then adjusted for the change in mass of the batch of field blanks. The sampling volume was calculated from the duration of sampling and the average of the start and end flow rates was recorded.

(1) $$\mathrm{Concentration}=\frac{\left(\mathrm{Average}\;\mathrm{post}\;\mathrm{weight}—\mathrm{Average}\;\mathrm{pre}\;\mathrm{weight}\right)\pm \Delta \;\mathrm{field}\;\mathrm{blank}}{\mathrm{Volume}}$$

The sampling schedule was based on the first 100 houses. There were no houses with metal roofs in this group, and succeeding homes were given the average value for urban or rural houses. The lower limit of detection (LOD) was calculated using 3 times the standard deviation of the weight change recorded in the field blanks (see Appendix A). Filters showing weight change of < LOD were assigned a value of one-half the LOD. Arithmetic and geometric means were calculated for each location in each season. These were then combined to produce an average for the year, by calculating a standardized mean, applying each sample a weight of 1 / (n × 3), where n = number of samples in that location in a season. The time-weighted average (TWA) was calculated using formula (2). The arithmetic mean concentration for each location was multiplied by the amount of time the child spent in it to produce the average exposure concentration over a 24 hour period.

(2) $$24-\mathrm{hour}\;\mathrm{TWA}=\frac{\sum {\mathrm{bt}}_{1}+{\mathrm{vt}}_{2}+{\mathrm{ot}}_{3}+{\mathrm{st}}_{4}+{\mathrm{ct}}_{5}+{\mathrm{kt}}_{6}\;}{24}$$

Where:

TWA: time weighted average
b: bedroom arithmetic mean
v: veranda arithmetic mean
o: outdoor arithmetic mean
s: school arithmetic mean
c: kitchen during cooking, arithmetic mean
k: kitchen outside cooking hours, arithmetic mean
t: time spent in each location

The average concentration was given on the DustTrak readout. This was converted to μg/m^3^ and a correction factor was applied, obtained from DustTrak-Apex side-by-side calibration: 0.43 for rural and 0.51 for urban samples (see Appendix B). A correction factor is required as the DustTrak is calibrated to “Arizona road dust”. The calibration factor would differ for different aerosols and other factors, such as relative humidity. The analysis was done using Excel (version 14.3.2, Microsoft Corp., USA), Prism (version 6.0a, Graphpad Software Inc., USA) and Stata (version 12.1; Stata Corp., USA).

### 2.7 Ethical approval

Ethical approval was granted by the Nepal Health Research Council and the UCL Research Ethics Committee. Written consent was taken at the first point of contact and verbal consent at each sampling time. As gratitude for taking part in the study, each family received a wall clock.

## 3 Results

### 3.1 Exposure data

In total, 291 microenvironment measurements were collected from 55 households (6.6% of the cohort), 8 representative outdoor locations, and 8 schools. Fifty-eight were discarded: in 14 cases repeated weighing produced differences greater than 0.1 mg, 13 had damage to the filter, in 15 cases there was equipment failure or inaccurate technique and in 16 cases there were mistakes in timing, labeling or location. Fifty-nine samples were below the LOD. In total, 110 days worth of air pollution data were gathered from across the various microenvironments, with a cumulative sampling duration of 2649 h. Table 1 shows the average duration and total time at each location.

Table 2 shows the concentration level by location in each season, and Table 3 shows average concentrations at each location over the year. In the bedroom, school and outdoors, concentration levels were the highest in winter, followed by spring/autumn, and the lowest in the monsoon season. The kitchen samples were lower in spring/autumn, possibly because more cooking is done outdoors and ventilation improves. In cold periods, fires are useful for heat. Veranda sample concentrations were the lowest in the monsoon season. The microenvironment measurements showed seasonal variation, with winter generally having higher concentration levels than spring/autumn, which were higher in turn than the monsoon season.

**Fig. 1:**
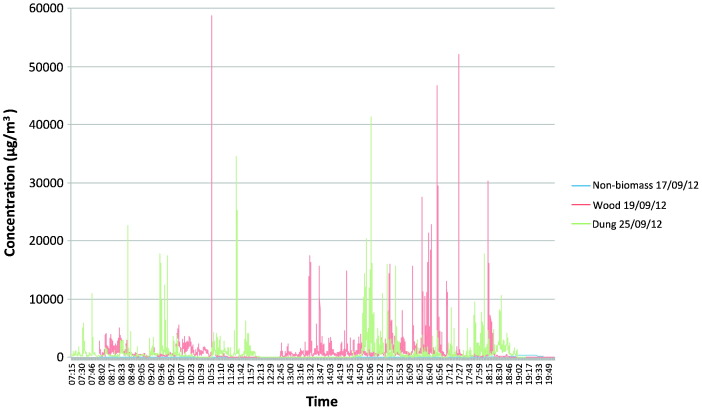
12-hour kitchen samples taken using the DustTrak II. The graph shows the concentration levels in three kitchens using non-biomass fuel, wood or dung as the main cooking fuel.

Seven DustTrak samples were taken in kitchens to look at variation in respirable fraction concentration over time. The two non-biomass samples had adjusted average (peak) concentrations of 53 μg/m^3^ (425 μg/m^3^) and 166 μg/m^3^ (1080 μg/m^3^). The biomass samples tended to have both average and peak concentrations approximately ten times higher. Both the dung and wood samples produced similar concentrations. The highest peak concentration was just below 60 000 μg/m^3^. Both dung samples produced similar concentrations, but the wood samples varied from an average of 167 μg/m^3^ to 831 μg/m^3^. Depending on kitchen structure, levels could rapidly come down to the non-cooking level, even in biomass-using houses. Levels in non-biomass households were many orders of magnitude lower than in biomass kitchens. A summary of the DustTrak data is shown in Appendix C, and time-concentration graphs from three kitchens are shown in Fig. 1.

**Fig. 2:**
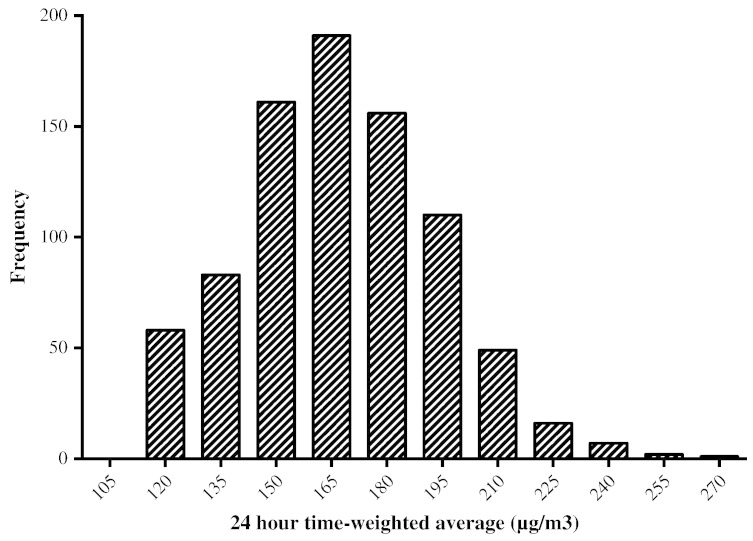
24-hour time-weighted average histogram, for all 834 children sample.

Table 4 shows the average exposure levels for children at each location, calculated by extrapolating the particulates in the samples from 55 representative households to the overall sample. The 24-hour TWA was modeled for 429 boys and 405 girls. No difference was found in exposure level between the sexes. The mean 24-hour TWA was 168 μg/m^3^ (SD = 25.9), again with no sex difference. The distribution of 24-hour TWA calculated for all children is shown in Fig. 2 and the average contribution of each location to the total concentration is shown in Table 4. The bedroom/living room made the largest contribution because of the duration of exposure. The veranda, bedroom and outdoors all made similar contributions, while the kitchen contribution was low.

### 3.2 Time activity

Questionnaire data were collected on 851 children (80.8%) out of a possible 1053. Three children had died and the data for 14 children were removed because they lived outside the region. The children were aged between seven and nine, with a mean age of 8.5. Time activity data are summarized in Table 4. Children spent most of their time indoors, amounting to about half the day on average. The amount of time at each location was similar for boys and girls. 2% of children did not go to school.

### 3.3 Cooking

Households used a variety of cooking fuels (Table 5). A different fuel was often used at the start of cooking or to light the fire. For example, straw and kerosene were commonly used at the start of cooking, but not as the main cooking fuel. About one-third of households used wood, one-third animal dung, and one-third gas, as their main fuels. It was common for families to alternate between fuels, usually with wood and dung. Animal dung cakes normally contained a small amount of wood or plant products to give them structure. The use of gas as a main fuel was more common in urban than rural settings (60.8% compared with 15.8%). The opposite was true for wood (27.2% compared with 41.7%) and dung (9.4% and 36.2%). Stoves were usually simple open fire designs of mud and clay with no chimney or hood. Cooking was done mainly indoors in the kitchen, and 786 (94%) households cooked indoors every day of the year.

### 3.4 House characteristics

Forty one percent of the sample lived in urban households. Houses had an average 3.6 rooms (range 1–15). Ninety-four households (11%) did not have a separate kitchen and 81 (10%) had only one room. Most house walls were of cement and brick (63%), with the remainder mostly mud and wattle (branches or sticks woven into the wall to add structure) (34%). Roofs were made from cement (43%), tiles (54%), grass or straw (2%), or metal sheet (1%). Half of the floors were cement or brick and the other half dirt.

## 4 Discussion

Our study is one of few to have examined children's exposure levels in a low resource rural setting in south Asia, and adds valuable information on their time activity. We found high exposure levels with substantial variability from season to season and by fuel type.

The mean 24-hour TWA of 168 μg/m^3^, and mean exposure levels in all locations, were very high compared with the World Health Organization recommendation of a maximum 24-hour mean of 25 μg/m^3^ for PM_2.5_ and 50 μg/m^3^ for PM_10_ (WHO, 2011) and also higher than the National Ambient Air Quality Standards for Nepal recommendation of 120 μg/m^3^ for PM_10_ (Central Bureau of Statistics, 2012b). Similar high microenvironment concentrations have been reported by others in the Himalayan region of northern India (Saksena et al., 1992), and Malawi (Fullerton et al., 2009). While our exposure levels were high, as shown by other research using comparable methods of exposure estimation, they were not unusual. Balakrishnan et al. found a higher mean 24 hour TWA PM_4_ exposure levels in children in Andhra Pradesh of 227–237 μg/m^3^ (Balakrishnan et al., 2004). In a study of PM_10_ 24 hour TWA in children in Bangladesh, similar mean levels of 156–196 μg/m^3^ were found (Dasgupta et al., 2006). Studies of PM_2.5_ exposure in children have estimated similar levels of 131–157 μg/m^3^ for 24 hour TWA from the Gambia (Dionisio et al., 2012). A further study of personal PM_2.5_ exposure in China, in which children carried the monitor with them where possible, found less than half the exposure level (46–70 μg/m^3^) (Baumgartner et al., 2011). These samples were taken only in the summer — in adults exposure was approximately double in the winter. If this were to be maintained for children, then these results would also be comparable to ours. There is limited information on indoor air pollution levels in Nepal. One study showed a higher geometric mean 24-hour level of 455 μg/m^3^ in houses where biomass fuels were burnt (Kurmi et al., 2013). Kurmi et al. also showed an approximately ten-fold higher concentration in rural households, attributed to biomass fuel burning, whereas in our study the levels were similar (Kurmi et al., 2008). These differences could be due to housing construction materials used in different climatic conditions and to types of fuel. Their study was carried out in a hilly region where the climate is colder and ventilation is minimal – whereas our study area had high temperatures in summer and well ventilated rooms – and households used hard wood rather than the mixed biomass in our sample. Our DustTrak:Apex calibration factors (Appendix B) were similar to those produced by Kurmi et al. (Kurmi et al., 2008).

The high peak levels recorded during cooking have been shown elsewhere in South Asia (Balakrishnan et al., 2011). Children were only in the kitchen for short periods but, as the extended 12-hour photometric sampling showed, they were exposed to extremely high peak levels with a great deal of variability from minute to minute during cooking: particulate concentrations at times reached almost 60 000 μg/m^3^. The incidence of respiratory infections seems to plateau with increasing particle mass over 1000–2000 μg/m^3^, suggesting that air pollution needs to be reduced to low levels to yield a health advantage (Ezzati and Kammen, 2001a,2001b).

Microenvironment sampling is superior to sentinel sampling in a few specific locations. We assumed that the bedroom had the same exposure level as any other room except the kitchen. Our time activity data showed children spent most of their time there and accounted for two fifths of total exposure. In most Nepalese households, rooms used for sleeping are also used for sitting in, to do homework or watch television. Contrary to our initial assumption, we did not find a large difference between different roof types, indicating that this was not the major source of ventilation.

While pollution emanates from the kitchen, where concentrations are high, its contribution to overall exposure was low because children spent little time there. This highlights the importance of time activity data. Ezzati and Kammen recommend using “time budgets” for this (Ezzati et al., 2000). We did not think that self-completed diaries would be possible as our participants were children and the levels of literacy were potentially low. Our questionnaire approach may have been prone to recall bias, in either direction, and the recall method does not distinguish seasonal change accurately enough. Other methods include direct observation, which is often considered the gold standard, and the use of Global Positioning System (GPS) monitoring. These methods were considered logistically infeasible, intrusive and not accurate enough to distinguish room location.

School, veranda and outdoor locations contributed similar proportions. To our surprise, concentration levels on the veranda were high in winter, spring and autumn seasons. The high exposure levels were likely to be from fires inside the house, outdoor fires and from other houses. The samples were taken in the early evening, which is the main cooking time. Dust may also be displaced from outdoors where levels were also fairly high, particularly in winter, from fires, fields and unsealed roads. Exhaust emissions would have made up a small proportion as mechanized traffic was relatively uncommon. Household air pollution is thought to contribute to about 16% of ambient air pollution worldwide (Lim et al., 2012). To a certain extent any source of particle mass is detrimental to health (Pope et al., 2009), and we are unable to quantify the source of particle mass without further work. It would be fair to say, however, that most indoor exposure arises from burning of biomass fuels, as indicated by the very high levels during cooking. Marked seasonal variation was seen in non-kitchen samples, with winter concentrations being higher than summer, which in turn were higher than in the monsoon. As expected, rain and moisture in the air reduce airborne particle mass in the monsoon season. This pattern was described by Saksena et al. (Saksena et al., 1992) and Baumgartner et al. who showed approximately a doubling of the concentration in the winter compared to the summer (Baumgartner et al., 2011).

Most households used biomass fuels. Animal dung is regarded as the least efficient and most polluting, the bottom rung of the WHO “energy ladder” (Rehfuess, 2006). Our data show an approximately three times higher proportion of households using gas than regional/national figures (Central Bureau of Statisics, 2012a), because of our semi-urban sample proportion, but most households used a mixture of fuels. Some used more expensive fuel to start cooking, or for snacks, and continued with a cheaper fuel. More expensive fuels like gas may be used for short cooking occasions like making tea and snacks, while biomass fuels are used for cooking main meals. Many households used two fuels, particularly dung combined with firewood or agricultural residues. This is related to supply issues such as availability and demand issues such as family income. Bates et al. found increased odds of infection related to not only biomass fuels, but also kerosene and gas usage, in comparison with electricity. This has important implications for our entire sample (Bates et al., 2013). As exposure levels varied greatly, from one fuel to another and at different points in the cooking process (Fig. 1), a more detailed description of exposure levels during cooking is required.

Women are known to experience higher exposure levels due to their role in cooking and female children have been thought to spend more time in the kitchen helping with cooking (Balakrishnan et al., 2002; Dasgupta et al., 2006; Ezzati et al., 2000; Saksena et al., 1992). Unlike others, we did not see a difference between boys and girls in time activity or exposure levels (Balakrishnan et al., 2002; Dasgupta et al., 2006; Ezzati et al., 2000; Saksena et al., 1992). It may be that the children in our study were too young to help with the cooking. Local staff have observed that boys are more exposed in their first two years, while girls start to help their mothers with cooking at around 10–12 years and will be more exposed during their early teens.

Our study had limitations. We assumed that the samples were representative of the true average concentration level. Sample variability was wide within one location and from one season to another. We cannot be certain that we captured all the variability. Gravimetric sampling over short periods in microenvironments where particulate levels are low is problematic. Filter weight-gains will often be small and results < LOD are of questionable utility (Helsel, 2010). While still advocating for the use of gravimetric sampling in general, for future sampling we would also recommend greater use of photometric devices where concentration levels are thought to be low or for short samples. Given the exposure levels found from the other samples, we feel that the non-detect values are likely to be lower, leading to an under-estimate of mean concentration. We would however stress the need for calibration with gravimetric sampling when photometric methods are used.

We were unable to quantify the amount of time a child was next to a fire, and how far away from the fire she was during cooking. The photometric data suggest that peak levels can be very high. Barnes et al. compared different methods of locating individuals and found that, while they were similar in terms of the time spent in a room, they differed in distance from a fire (Barnes et al., 2005). More detailed descriptions of housing construction materials and fuel use, combined with measurements and recordings, would be needed to take this further.

## 5 Conclusion

Our study quantified the particle mass levels to which children are regularly exposed in rural villages of Nepal. While further studies are needed to verify our findings, they point to potentially important interventions. In the real world, there are major obstacles to reducing exposure. Changing fuel usage and improving ventilation are complicated and costly and sometimes face social and cultural obstacles. Children of this age tend to spend a short time in the kitchen and veranda, but both locations are associated with high exposure levels. If children could be in a clean environment at critical times of the day, such as early evening when cooking is taking place, their exposure would be reduced.

## Role of the funding source

The sponsor had no role in the study design, data collection, analysis, interpretation or writing of the article. DD had access to all study data and responsibility for the decision to submit for publication.

## Funding

The research was funded by the Wellcome Trust (grant number 092121/10/Z). The funder played no part in the design, analysis, or reporting of the study.

## Conflict of interest statement

The authors declare no conflict of interest.

## Author contributions

The study was designed by D Devakumar, D Osrin and J Ayres. D Devakumar oversaw the study, with technical support provided by S Semple and OP Kurmi. SK Yadav coordinated the data collection. B Shrestha, DS Manandhar, N Saville and A Costello provided overall supervision and management of the field site and staff. D Devakumar wrote the first draft. All authors were involved in data interpretation, read and criticized drafts of the manuscript.

## Figures and Tables

**Table 1:** Sampling times and duration.

Location	Number of samples	Average start time (range)	Average end time (range)	Average duration (minutes)	Total sampling time (hours)
Bedroom	96	15:49 (14:30 to 17:38)	09:35 (07:52 to 15:25)	1064 (854 to 1397)	1720
Veranda	31	16:53 (15:43 to 17:05)	19:53 (18:42 to 20:05)	180 (179 to 180)	96
Kitchen cooking	31	07:48 (07:00 to 08:50)	10:38 (09:20 to 11:59)	163 (60 to 202)	85
Kitchen no cooking	29	11:43 (09:41 to 15:04)	14:18 (13:17 to 18:04)	184 (180 to 229)	92
Kitchen 12 hour samples	7	07:24 (07:00 to 08:00)	19:24 (19:00 to 20:00)	720 (720 to 720)	96
School	22	09:26 (06:55 to 11:57)	13:55 (11:15 to 16:30)	275 (120 to 330)	101
Outdoors	38	07:11 (05:07 to 10:30)	19:02 (17:07 to 20:24)	725 (678 to 796)	459

**Table 2:** Respirable particle mass concentration by season in different locations.

Location			Winter		Spring & autumn				Monsoon	
		Number of samples	Arithmetic mean concentration (SD) (μg/m^3^)	Geometric mean concentration (SD) (μg/m^3^)	Number of samples	Arithmetic mean concentration (SD) (μg/m^3^)	Geometric mean concentration (SD) (μg/m^3^)	Number of samples	Arithmetic mean concentration (SD) (μg/m^3^)	Geometric mean concentration (SD) (μg/m^3^)
Bedroom	Urban cement roof	16	342 (185)	303 (1.64)	10	113 (96.9)	79.6 (2.60)	4	60.3 (35.4)	49.7 (2.24)
	Urban tiled roof	4	384 (234)	311 (2.33)	4	112 (50.8)	102 (1.73)	4	204 (182)	123 (4.05)
	Urban straw roof	1	256	256	2	62.9 (67.4)	41.1 (4.06)	1	38.5	38.5
	Rural cement roof	8	322 (109)	307 (1.38)	7	153 (95.3)	115 (2.69)	4	115 (68.5)	85.1 (2.97)
	Rural tiled roof	16	398 (216)	352 (1.65)	4	173 (201)	111 (2.78)	2	66.4 (71.3)	43.1 (4.09)
	Rural straw roof	2	217 (1.80)	217 (1.01)	4	374 (304)	285 (2.39)	3	160 (249)	48.8 (6.83)
Veranda	Urban	8	410 (221)	347 (1.98)	2	850 (460)	786 (1.77)	5	58.0 (28.2)	50.3 (1.95)
	Rural	8	771 (1060)	455 (2.72)	2	469 176	452 (1.47)	6	95.0 (63.2)	78.5 (1.99)
School	Urban	5	167 (86.8)	146 (1.85)	4	115 (74.3)	98.4 (1.89)	5	82.8 (45.1)	75.8 (1.54)
	Rural	2	161 (143)	126 (2.83)	4	63.1 (8.93)	62.6 (1.16)	2	71.8 (3.30)	71.8 (1.05)
Outdoor	Urban	7	305 (216)	259 (1.79)	7	105 (67.1)	85.0 (2.15)	5	24.3 (0.32)	24.4 (1.01)
	Rural	5	369 (207)	319 (1.90)	8	112 (66.5)	99.6 (1.65)	6	67.6 (27.6)	61.6 (1.66)
Kitchen cooking	Biomass	7	1550 (1050)	1311 (1.84)	5	433 (368)	318 (2.46)	9	933 (499)	835 (1.63)
	Non-biomass	2	204 (170)	165 (2.60)	5	97.8 (2.33)	97.8 (1.02)	3	177 (147)	143 (2.15)
	Dung	4	1410 (757)	1270 (1.70)	2	557 (649)	315 (5.23)	4	1140 (686)	982 (1.89)
	Wood	2	2270 (1740)	1910 (2.36)	2	244 (47.7)	242 (1.22)	4	775 (304)	730 (1.49)
Kitchen no cooking	Biomass	10	250 (128)	216 (1.85)	4	707 (1220)	216 (5.17)	7	96.0 (2.63)	95.9 (1.03)
	Non-biomass	4	123 (45.7)	117 (1.45)	4	303 (416)	168 (3.12)			
	Dung	4	260 (68.1)	251 (1.36)	3	95.2 (2.92)	95.2 (1.03)	2	92.9 (3.44)	92.8 (1.04)
	Wood	6	234 (168)	183 (2.30)	1	2550	2550	4	97.1 (1.05)	97.1 (1.01)
Total		106			76			66		

NB: Biomass samples are shown together and separated into those that used wood and dung only (the others used a mixture of the two).

**Table 3:** Weighted average respirable particle mass over the year, calculated by applying a weighting to each sample of 1 / (n × 3), where n = number of samples in that location in a season.

Location		Number of samples	Standardized mean (μg/m^3^)	95% Confidence interval
Bedroom	Urban cement roof	30	116	87.8 to 144
	Urban tiled roof	12	233	133 to 333
	Urban straw roof	4	134	109 to 159
	Rural cement roof	19	175	134 to 217
	Rural tiled roof	22	125	50.6 to 199
	Rural straw roof	9	236	120 to 351
	Combined roof urban	47	166	117 to 215
	Combined roof rural	50	192	135 to 249
Veranda	Urban	15	592	281 to 902
	Rural	16	445	264 to 627
School	Urban	14	121	82.4 to 160
	Rural	8	123	37.5 to 209
Outdoors	Urban	19	131	80.7 to 181
	Rural	19	202	128 to 276
Kitchen cooking	Biomass together	21	908	614 to 1203
	Non-biomass	10	175	63.2 to 286
Kitchen no cooking	Biomass	21	438	− 138 to 1010
	Non-biomass	8	213	− 15.8 to 442

**Table 4:** Time activity and exposure level to respirable particulates by location, for girls and boys. Exposure levels were calculated by multiplying average concentrations in each location by the time each child was in it.

Location		Time activity				Exposure				
		Mean (hours)	Standard deviation	Minimum (hours)	Maximum (hours)	Mean (μg/m^3^)	Standard deviation	Minimum (μg/m^3^)	Maximum (μg/m^3^)	Proportion of total contribution (%)
Bedroom/living room	Boys	12.2	1.65	7.5	17	1750	530	936	3500	44
	Girls	12.3	1.77	8	18	1800	506	998	3730	44
Veranda	Boys	1.24	1.10	0	5.5	600	540	0	3250	15
	Girls	1.33	1.23	0	5.5	640	603	0	3250	16
Kitchen during cooking	Boys	0.04	0.23	0	2	20.2	147	0	1820	1
	Girls	0.03	0.19	0	2	19.7	136	0	1820	1
Kitchen when there is no cooking	Boys	0.21	0.53	0	4	66.7	180	0	1310	2
	Girls	0.21	0.49	0	3	71.0	170	0	875	2
Outdoors	Boys	5.07	1.64	1	12.5	915	369	196	2420	23
	Girls	4.83	1.56	0	12	884	355	196	2420	22
School	Boys	5.29	1.01	0	10	647	123	0	1210	16
	Girls	5.26	1.04	0	11	644	128	0	1330	16
Total concentration in 24 h	Boys					4000	607	2850	6120	
	Girls					4060	636	2830	6440	
24 hour time-weighted average	Boys					167	25.3	119	255	
	Girls					169	26.5	118	268	

**Table 5:** Fuel usage. The number of households using a fuel to light the fire and during cooking is shown. In both these cases, multiple fuels may be used by the household. If more than one fuel was used, the main fuel used for cooking was also recorded.

Fuel type	Fuel used to light the fire	Fuel used continuously	Main fuel used by a household (%)
Wood	7	606	309 (37.1)
Dung	0	392	231 (27.7)
Straw	138	10	6 (0.7)
Charcoal	2	3	4 (0.5)
Other plant products	14	34	21 (2.5)
Kerosene	230	6	3 (0.4)
Gas	241	324	251 (30.0)
Biogas	7	8	8 (1.0)
Electricity	3	4	1 (0.1)
Plastic	10	0	0
Paper	4	NA	NA
Matches	9	NA	NA
