# Supplementary material for: Biomass fuel use and the exposure of children to particulate air pollution in southern Nepal
Source: Environ Int. 2014 May;66(100):79–87. doi: 10.1016/j.envint.2014.01.011 (PMC3989062; doi:10.1016/j.envint.2014.01.011)
Supplement: Supplementary file 1 — Supplementary tables. [file mmc1.docx]

|  | Change in field blanks | Limit of detection |
| --- | --- | --- |
| Batch 1 | -0.000020 | 0.000069 |
| Batch 2 | -0.000017 | 0.000072 |
| Batch 3 | 0.000033 | 0.000077 |
| Batch 4 | 0.000015 | 0.000076 |

**Appendix A: Change in mass of field blanks and Limit of detection value**

|  | DustTrak II concentration (µg /m^3^) | Apex concentration (µg /m^3^) | DustTrak:Apex  ratio | Average correction factor |
| --- | --- | --- | --- | --- |
| Rural | 1670 | 732 | 0.4387 | 0.43 |
|  | 1590 | 669 | 0.4211 |  |
| Urban | 1370 | 660 | 0.4897 | 0.52 |
|  | 957 | 509 | 0.5438 |  |

Appendix B: DustTrak II correction factor. Two urban and two rural samples were taken with both the DustTrak II and the Apex to calculate a site-specific ratio for photometric:gravimetric sample conversion.

|  | Length of sample | Fuel used | Minimum concentration  (µg/m^3^ ) | Maximum concentration  (µg/m^3^ ) | Average concentration  (µg/m^3^ ) |
| --- | --- | --- | --- | --- | --- |
| 1. | 12 hours | Non biomass | 0 | 425 | 53.0 |
| 2. | 12 hours | Non biomass | 53.6 | 1080 | 166 |
| 3. | 12 hours | Wood | 26.0 | 58 800 | 831 |
| 4. | 12 hours | Wood | 0 | 1930 | 167 |
| 5. | 12 hours | Wood | 40.6 | 10 500 | 498 |
| 6. | 12 hours | Dung | 58.5 | 41 400 | 684 |
| 7. | 12 hours | Dung | 0 | 52 000 | 718 |

Appendix C: DustTrak II 12-hour kitchen samples, showing the minimum, maximum and average concentrations in each location.
